# Evaluating the Antimicrobial Efficacy of a Designed Synthetic Peptide against Pathogenic Bacteria

**DOI:** 10.4014/jmb.2405.05011

**Published:** 2024-08-31

**Authors:** Maria Caroline de Moura Cavalheiro, Caio Fernando Ramalho de Oliveira, Ana Paula de Araújo Boleti, Layza Sá Rocha, Ana Cristina Jacobowski, Cibele Nicolaski Pedron, Vani Xavier de Oliveira Júnior, Maria Lígia Rodrigues Macedo

**Affiliations:** 1 Protein Purification Laboratory and its Biological Functions; Faculty of Pharmaceutical Sciences, Food and Nutrition; Faculty of Pharmacy, Food and Nutrition; Federal University of Mato Grosso do Sul (UFMS), Campo Grande, Mato Grosso do Sul, Brazil; 2 Center for Natural and Human Sciences of the Federal University of ABC (UFABC), São Paulo, SP, Brazil

**Keywords:** Bacterial resistance, biofilm, drug design

## Abstract

Recent research has focused on discovering peptides that effectively target multidrug-resistant bacteria while leaving healthy cells unharmed. In this work, we describe the antimicrobial properties of RK8, a peptide composed of eight amino acid residues. Its activity was tested against multidrug-resistant Gram-negative and Gram-positive bacteria. RK8's efficacy in eradicating mature biofilm and increasing membrane permeability was assessed using Sytox Green. Cytotoxicity assays were conducted both in vitro and in vivo models. Circular dichroism analysis revealed that RK8 adopted an extended structure in water and sodium dodecyl sulfate (SDS). RK8 exhibited MICs of 8-64 μM and MBCs of 4-64 μM against various bacteria, with higher effectiveness observed in Methicillin-resistant *Staphylococcus aureus* (MRSA) and *E. coli* KPC+ strains than others. Ciprofloxacin and Vancomycin showed varying MIC and MBC values lower than RK8 for Gram-positive bacteria, but competitive for Gram-negative bacteria. The combination of RK8 and ciprofloxacin showed a synergistic effect. The RK8 peptides could reduce 38% of the mature *Acinetobacter baumannii* biofilm. Sytox Green reagent achieved 100% membrane permeation of Gram-positive and Gram-negative bacteria. The RK8 peptide did not show cytotoxic effects against murine macrophages (64 μM), erythrocytes (100 μM) or *Galleria mellanella* larvae (960 μM). In the stability test against peptidases, the RK8 peptide was stable, maintaining around 60% of the molecule intact after 120 min of incubation. These results highlight the potential of RK8 to be a promising strategy for developing a new antimicrobial and antibiofilm agent, inspiring and motivating further research in antimicrobial peptides.

## Introduction

The recent surge in multidrug-resistant bacteria, a pressing issue in public health, directly results from the overuse of antibiotics and the sluggish pace of antimicrobial drug development [1]. While discovering antimicrobials has historically been a life-saving breakthrough, the persistent challenge of multi-resistance infections underscores the urgent need for new, less toxic, and more effective antimicrobials [2-4].

A new proposal for new drug development is antimicrobial peptides (AMPs). These molecules act as effective antibiotics, whose antimicrobial activity results from disrupting the cell membrane integrity. Unlike conventional antimicrobial agents, AMPS acts on multiple targets within the cell [5]. Most antibiotics interact with and inhibit specific biochemical processes in bacteria, such as cell wall synthesis (peptidoglycan), protein synthesis (translation), DNA replication, RNA synthesis (transcription), and folic acid biosynthesis. However, some antibiotics interfere with ion channels and cause bacteriolysis [6]. AMPs exert their activity mainly through the rupture of the bacterial cell membrane, with consequent cell death or immunomodulatory actions [7].

AMPs have garnered significant interest as potential antibiotics for the future. Cellular organisms internally produce these small molecules, encompassing 2-50 amino acids and are essential components of the host's natural immune response. Their small size and diverse mechanisms of action, which impede the development of bacterial resistance, make them a promising alternative to conventional therapeutics. AMPs also display rapid bactericidal action, independent of phenotypic resistance, and good solubility in water with thermal stability [8, 9].

Their cationic and amphiphilic properties drive AMP antibacterial activity. It presents a variable net positive charge and about 50% hydrophobic amino acids. These peptides establish electrostatic interactions with anionic bacterial membranes, rupturing them. Their hydrophobic and hydrophilic properties facilitate interaction with the lipid tails of lipopolysaccharide (LPS) and the hydrophilic heads of phospholipids in the bacterial membrane. For this reason, it compromises the membrane permeability and leads to bacterial cell death [9]. There is significant interest in researching natural products despite their limitations. Applying synthetic biology can overcome production challenges and enhance chemical diversity. Computational tools assist in peptide sequence design and structure with bactericidal action. The broad-spectrum in vitro, in vivo and silico antibacterial activity of AMPs offers a promising alternative to conventional therapeutics [10].

Our research involved the development of the RK8 peptide using computer-aided methods to leverage its physicochemical properties in investigating its antibacterial activity and cell selectivity. The peptide sequence comprises eight amino acid residues and has a molecular mass of 1,273.54 Da, net charge +5, and hydrophobic ratio of 38%. Its amino acid residues do not fold into regular secondary structure elements. Therefore, we investigated its antimicrobial, antibiofilm, and toxicity properties in the present study. In addition, our study has illustrated the synergistic interaction of RK8 in combination with ciprofloxacin, along with its non-toxic attributes in both in vitro and in vivo settings. Finally, we also determined that its mechanism of action involves membrane damage. However, it acts differently on Gram-negative and Gram-positive membranes.

## Material and Methods

### Peptide Design and Synthesis

Based on the structural characteristics shared by arginine- and tryptophan-rich peptides, our researchers created the RK8 peptide sequence. As our aims involved the synthesis of an ultrashort peptide, we started with a sequence of eight amino acid residues containing arginines, lysines, and tryptophan. We defined a net electrical charge of +5 to secure the sequence distributed between two arginines and three lysines. Three tryptophan residues influence 37.5% of the hydrophobic amino acid composition. The molecular structure was carefully devised to incorporate specific amino acid residues, allowing it to exhibit concurrently hydrophilic and hydrophobic properties. The Antimicrobial Peptide Database (APD3) and the PepDraw server (http://pepdraw.com/) had the resulting RKWRKWWK sequence submitted and analysed. Physicochemical parameters such as isoelectric point, molecular weight, and side chain arrangement were also evaluated [11].

Aminotech, in Diadema-SP, carried out the peptide synthesis. A synthesis > 95% was requested. Mass spectrometry (ESI-TOF) and high-performance liquid chromatography (HPLC) evaluated molecular mass and purity parameters. The concentration of the RK8 peptide in solution was determined by determining the absorbance at 280 nm, based on the extinction coefficient of the tryptophan residue (ε = 5.560 mol/ml). RK8 was solubilised in ultrapure water, and its absorbance was measured using an Evolution 201 spectrophotometer (Thermo Fisher Scientific, USA) at 280 nm (A280). To determine its concentration (C), the following formula was used:

C (mg/ml) = (A280 × FD × PM) / ε

In Daltons, FD is the dilution factor, and PM is the molecular weight of the peptide.

### Modelling and Validation

The peptide's secondary structure was determined using the APPTEST server (https://research.timmons.eu/apptest), which interactive simulations complete atomic models [12]. After modelling, we performed validation using PROCHECK software (https://www.ebi.ac.uk/thorntonsrv/software/PROCHECK/) to verify the stereochemistry and atoms' spatial arrangement of the peptide.

### Circular Dichroism

CD analyses were performed with a Jasco J-1100 spectropolarimeter (Jasco Inc., Japan) using a quartz cuvette with a 0.1 cm optical path. RK8 peptide was synthesised at a concentration of 30 μM in an aqueous solution and subjected to analysis in ultra-pure water in the presence of SDS (sodium dodecyl sulfate). The experiment involved six scans at 25°C, covering wavelengths from 190 to 280 nm. As part of the peptide preparation process, we gathered spectra of the used solutions, referring to them as blank solutions. We collect the resulting data after subtracting the implemented spectra from the blank solution. Afterwards, the data was transformed into residual molar ellipticity [θ] using the following equation:

[θ] = *θ* 10 * C * *l* * *n*r,

Where θ is the ellipticity measured in milliseconds, C is the concentration in molar units, l is the path length in cm, and nr is the number of amino acid residues.

### Microorganisms

To determine the antimicrobial activity of the RK8 peptide, bacteria purchased commercially and catalogued in the American Type Culture Collection (ATCC, USA) were used: Gram-negative: *Acinetobacter baumannii* ATCC 19906, *Escherichia coli* ATCC 35218, *Klebsiella pneumoniae* ATCC 700603, and *Pseudomonas aeruginosa* ATCC 27853; Gram-positive: *Staphylococcus saprophyticus* ATCC 29970.

Strains of multidrug-resistant clinical isolates were tested: Gram-negative *Acinetobacter baumannii* (IC 003321216) and the producer of carbapenemases *E. coli* KPC+ (IC 001812446); Gram-positive *Staphylococcus aureus* methicillin-resistant (MRSA) ATCC 33591 and ATCC 43300.

### Minimum Inhibitory Concentration (MIC) and Minimum Bactericidal Concentration (MBC) Assays

Clinical and Laboratory Standards Institute (CLSI) guidelines performed the peptide MIC assays. Bacterial colonies were isolated on the Muller Hinton Agar (MHA) and plated at a final concentration of 1.5 × 10^5^ CFU/ml. The peptides were tested at concentrations ranging from 0.5 μM to 32 μM. Antibiotic control, positive control, was carried out using 10 μl of ciprofloxacin (for targeting Gram-negative bacteria) or vancomycin (for targeting Gram-positive bacteria) at a concentration of 1 μM, in combination with 90 μl of MH culture medium.

The term "MIC" refers to the minimum inhibitory concentration of a peptide, which is the lowest concentration capable of reducing bacterial growth by 90% or more. For the results, bacterial growth was determined by visual inspection and confirmed by absorbance measurement at 595 nm after 24 h of incubation using a Multiskan GO microplate reader (Thermo Fisher Scientific, USA). For assays involving resistant strains such as MRSA strains and clinical isolates of *E. coli* and *A. baumannii*, we used an initial inoculum at 0.5 McFarland directly in the microplates to establish the control MIC with a higher bacterial load. The rest of the assay was conducted as previously described.

The MBC was determined based on the MIC results. Three replicates of 10 μl were extracted from the microplate wells, placed on MHA, and then kept in an incubator at 37°C for 24 h. The MBC was determined to have the lowest peptide concentration, and no bacterial growth was detected. All experiments were performed in triplicate.

### RK8 and Ciprofloxacin Synergistic Effect

An investigation of the potential synergistic effect of combining the RK8 peptide with the quinolone ciprofloxacin antibiotic was conducted. The assay was performed in a 96-well microplate, where the two drugs, RK8 and ciprofloxacin, were present. Serial dilution was undertaken to establish a range of concentrations aligned with their corresponding minimum inhibitory concentration (MIC) values. Only the multidrug-resistant strains *A. baumannii* (IC 003321216) and *E. coli* KPC+ (IC 001812446) were used in these assays. Detection of *Klebsiella pneumoniae* carbapenemase (KPC), an enzyme commonly found in Enterobacteriaceae bacteria, is a significant marker of antibiotic resistance.

An assessment of synergistic effects was conducted using synergy scoring models on the SynergyFinder platform (https://synergyfinder.org/). The models included Highest Single Agent (HSA), Loewe Additivity, Bliss Independence, and Zero Interaction Potency (ZIP). Loewe Additivity is a model that assumes that drugs with similar modes of action produce an additive effect, identifying synergy if the observed effect exceeds this additive expectation. These models compare the observed combination effect to expected outcomes, identifying synergy when the observed effect exceeds the predicted effect based on each model's assumptions. We used the Bliss model to calculate the synergistic impact of RK8 in combination with ciprofloxacin. This model assumes that the two drugs exert their effects independently, and the expected combination effect can be calculated based on the probability of independent events [13].

yB L ISS = y1+y2-y1·y2

RK8 concentrations ranging from 0 to 16 μM were combined to assemble the concentration matrix with ciprofloxacin concentrations ranging from 0 to 32 μM. For the analyses carried out with *E. coli* KPC+, an initial concentration of 32 uM was used, while for *A. baumanni*, we used an initial concentration of 128 μM.

### Membrane Permeability

Membrane permeability was investigated as described by Mohanram & Bhattacharjya (2016), and Almeida *et al*. proposed modifications [14]. In the assay, *E. coli* (ATCC 35218) and MRSA (ATCC 43300) strains suspension grown in MH broth for 18 h at 37°C. Afterwards, a phosphate buffer with OD_600_ nm at 0.5 in 10 mM sodium, pH 7.0, was prepared. Then, 280 μl of the bacterial suspension was transferred to 96-well black microplates, where 10 μl of Sytox Green at 30 μM was added and incubated for 10 min at 37°C. Subsequently, 10 μl of the peptide RK8, at a concentration 30 times the MIC, was added to each well, and the kinetic assay was performed for 50 min, with readings every 5 min. The assay was performed with fluorescence readout, excitation at 485 nm, and emission at 520 nm in a Varioskan Lux microplate reader (Thermo Fisher Scientific). The negative control of membrane damage was performed with *E. coli* KPC+001812446 incubated with 10 μl of 10 mM sodium phosphate buffer, pH 7.0. Three independent experiments were performed in triplicate [14, 15].

### Biofilm Activity

The RK8 eradication effect on mature *A. baumannii* biofilm (IC 003321216) was investigated. For this purpose, sterilised 11 mm circular coverslips were placed in 24-well microplates. The plates containing the coverslips were UV sterilised for two hours. Then, the coverslips received 500 μl of a sodium bicarbonate solution containing 20%human plasma. Human plasma was previously collected in tubes containing EDTA and was used to stimulate the adherence of bacteria to the glass [16].

The microplate was incubated with the bicarbonate plasma solution overnight at 4°C. The following day, the wells were aspirated and washed once with 500 μl of saline. A culture of *A. baumannii* IC 003321216, cultivated overnight in Brain Heart Infusion (BHI) medium supplemented with 0.4% glucose, was resuspended in saline to an OD600 of 0.2. Subsequently, it was diluted 100-fold in a BHI medium containing 0.4% glucose. Aliquots of 500 μl of the suspension were distributed across the pre-treated coverslips. The microplate was incubated under constant agitation (100 rpm) at 37°C for 24 h to form the biofilm.

After biofilm formation, the wells were washed twice with a BHI medium containing 0.4% glucose to remove non-adherent bacteria. They received 500 μl of the medium used in the assay containing RK8 at the MIC and half this concentration (MIC /two). Biofilm control received only a BHI medium containing 0.4% glucose, and control with the antibiotic ciprofloxacin at MIC (128 μM) was also performed. The biofilm was incubated under constant agitation (100 rpm) at 37°C for 24 h to analyse the effect of RK8 on eradicating the mature form of the *A. baumannii* biofilm. At the end of the 24-h incubation period, the wells were washed twice again with a BHI medium containing 0.4% glucose to remove non-adherent bacteria. At this stage, three wells received 500 μl of the MTT solution at 0.5 mg/ml to determine biofilm viability.

The plate was incubated at 37°C for 30 min, the culture medium was aspirated, and the reduced formazan crystals were dissolved with Dimethyl sulfoxide (DMSO). Three aliquots from each of the wells were transferred to a microplate (*n* = 9), where the absorbance was detected at 630 nm. A fourth well of the 24-well microplate was used to capture bloom images using a Leica DM 2000 LED microscope using 20x magnification. The biofilm coverslips were washed with saline and incubated with the dyes SYTO9 and propidium iodide, present in the LIVE/DEAD BacLight Bacterial Viability Kit (Invitrogen, USA). The reagents were diluted in physiological saline, in the proportion of 1μl of dyes for each 1 ml of serum. Each coverslip was incubated with 500 μl of the staining solution for 10 min in the dark. Excess dye was removed by washing in saline solution, and the coverslips were mounted on a microscopic slide and observed. Representative viability images determined with MTT were taken using the filters for red and green colours.

### Cytotoxic Effects

**Hemolytic activity.** As hemolysis is an effect widely observed among antimicrobial peptides, we investigated the RK8 peptide hemolytic activity based on the protocol proposed by Uggerhøj *et al*. using erythrocytes [17]. The blood sample (1 ml) was washed three times with 3 ml of saline solution through centrifugation cycles of 8 min at 1,380 ×*g* to secure a concentrated solution of erythrocytes. A 1% erythrocyte solution was meticulously prepared and subsequently transferred to microtubes containing 200 μl. The solution was incubated with 200 μl of the RK8 solution (1.56-200 μM). Thus, the concentration of RK8 evaluated ranged from 100 to 0.78 μM. The hemolysis positive control was performed with 0.06% (v/v) Triton X-100 solution and the negative control with saline solution. The samples were incubated for 60 min at 37°C and sequentially centrifuged for 10 min at 2,455 ×*g*. Subsequently, 100 μl of the supernatant was transferred to 96-well microplate wells in triplicate. The free haemoglobin content was determined at 414 nm on a Multiskan GO microplate reader (Thermo Fisher Scientific).

**Cellular viability.** RAW 264.7 murine macrophages' cellular viability was evaluated in the presence of the RK8 peptide, according to the method proposed by Mosmann [18], using the enzymatic reduction of the reagent 3-(4,5-dimethylthiazol-2-yl bromide) -2,5-diphenyltetrazolium (MTT). The cells were provided by Octávio Franco (Dom Bosco Catholic University - UCDB) and cultivated in Dulbeccós Modified Eagle Medium - high glucose (DMEM), supplemented with 10% Fetal Bovine Serum (FBS), 100 U/ml of penicillin and 100 μg/ml of streptomycin (Gibco, Brazil), at 37°C in an incubator at 5% CO_2_.

After detaching from the culture bottle, their density was determined, and they were seeded in a 96-well microplate at a density of 6 × 10^3^ cells per well. After being placed in microplates, the cells were incubated for 24 h. The culture medium was replaced with a DMEM culture medium lacking SFB supplementation. Instead, it contained a serial dilution of RK8 ranging from 1 to 64 μM. After 24 h, the medium containing RK8 was removed and replaced with 100 μl of DMEM medium containing MTT 0.5 mg/ml. After four hours of incubation, the medium was removed again, and the reduced formazan crystals were resuspended with the addition of 100 μl of DMSO. Then, the absorbance of the wells was determined at 630 nm in a Varioskan microplate reader (Thermo Fisher Scientific). Three independent experiments were performed in triplicate. Cell viability was calculated from the following formula:

Cell viability (%) = (Abs_Sample_÷Abs_Negative control_) × 100

***Galleria mellonella* in vivo toxicity.** The RK8 sample acute toxicity was determined in an in vivo model using *G. mellonella* larvae. In this test, ten larvae (*n* = 10) of *G. mellonella* weighing between 200 and 300 mg were selected for each group. The RK8 solution, at concentrations of 16 μM, 160 μM, and 960 μM (equivalent to MIC, 10 MIC, and 60 MIC, respectively), was administered to the hemocoel of each larva through the penultimate left paw using a Hamilton syringe. Four groups of larvae were divided; the first group was used as a negative control, applying 10 μl of 0.9% saline solution to their hemocoel. Three different concentrations of RK8 diluted in 0.9%saline were applied in the other groups. Larvae were incubated at room temperature, and their survival was recorded at time intervals for up to 72 h.

### Peptide Stability

The RK8 peptide stability was evaluated using a 25% FBS solution prepared in ultrapure water. This assay was performed by adding 40 μl of the peptide solution (10 mg/ml) to 1 ml of 25% FBS solution. The mixture was maintained at 30°C. Aliquots of 150 μl were collected after 0, 15, 30, 45, 60, and 120 min, mixed with ten μl of Trifluoroacetic Acid (TFA) and centrifuged at 14 000 rpm at 4°C for 10 min [19]. High-Performance Liquid Chromatography (HPLC) monitored the stability of the peptide using the C-18 reversed-phase hydrophobic column (μ Bondapak C18, 3.9 × 300 mm, Waters, USA) at 25°C. The peptide's remaining percentage during degradation kinetics was calculated by integrating the peak area corresponding to the RK8 peptide, injecting 40 μl of the incubation product in triplicate. The assay was conducted in an Ultimate 3000 chromatograph (Thermo Fisher Scientific). A separation program of 60 min at 1 ml/min was implemented, with monitoring of the 220 and 280 nm wavelengths. Thermo Scientific's ChromeleonTM Chromatography Data System (CDS) 7 software determined the area under the curve.

### Statistical Analysis

The experimental results showed that statistical significance was determined by a one-way Student's *t*-test or a one-way analysis of variance (ANOVA) followed by Dunnett's test. Values of *p* < 0.05 were considered statistically significant. GraphPadPrism version 8.0 was used for all statistical analyses.

## Results

### RK8 Peptide Racional Design

**Fig. 1:**
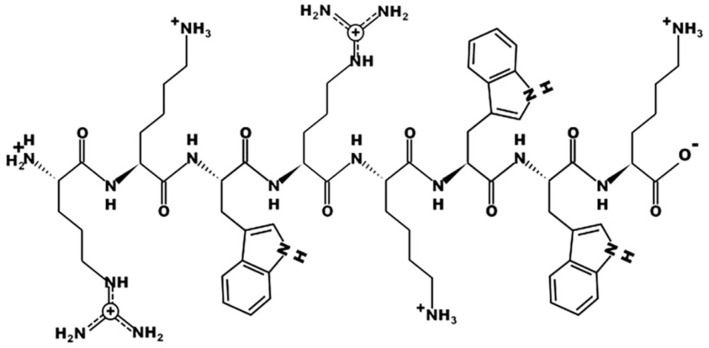
Primary structure of the RK8 peptide, collected through the PepDraw server.

RK8 sequence (RKWRKWWK) has eight amino acid residues, +5 net charge, and hydrophobicity of 38%, with the first residue being arginine (R) and the eighth being lysine (K) (Table 1). Peptide sequence was created from a two sequences combination: 2 repetitions of a three amino acids sequence: Arg–Lys–Trp, occupying positions 1 to 6, and an inverted repeat of residues 5 and 6 (Lys–Trp), giving rise to amino acids at positions 7 and 8 (Trp–Lys). The structural design was produced to ensure positively charged residues at the amino and carboxyl termini. The physicochemical properties and primary structure of RK8 were collected from the APD3 database and the PepDraw platform. To validate this, PROCHECK software was used to confirm the chiral centres and ensure no steric hindrances in the correct orientation (Fig. 1).

### Circular Dichroism

**Fig. 2:**
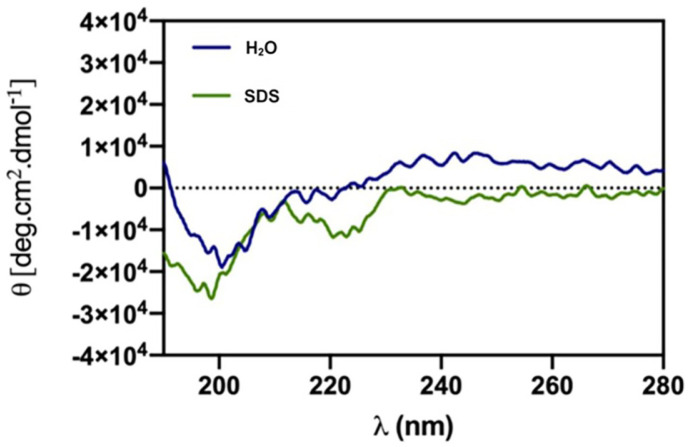
Circular dichroism spectra of the RK8 peptide at 30 μM, in both water and SDS at 25°C.

After synthesising, we characterised the RK8 secondary structure through Circular Dichroism (CD) studies. The results exhibit RK8 dissolution in water and 30 mM SDS. RK8 exhibits a random structure in both solutions, with low-magnitude negative bands at 200 nm (Fig. 2), which suggests that the peptide does not adopt an alpha-helical conformation. Therefore, we speculate that the peptide has an extended conformation.

### Antibacterial Activity

The MIC was determined using the broth microdilution method (CLSI, 2012), using different concentrations of RK8 against susceptible and resistant bacteria. For Gram-positive bacteria, RK8 exhibited MIC values ranging from 8 μM to > 64 μM and MBC values from 8 μM to 64 μM. Ciprofloxacin showed MIC values between 1 μM and four μM, with corresponding MBC values of 1 μM to 8 μM. Vancomycin had consistent MIC and MBC values of 1μM for *Staphylococcus aureus* (MRSA) strains, while values for *Staphylococcus saprophyticus* were 0.5 μM for MIC and one μM for MBC. For Gram-negative bacteria, RK8's MIC values ranged from 16 μM to > 64 μM and MBC values from 1 μM to 64 μM. Ciprofloxacin displayed MIC values from 0.5 μM to 128 μM, with MBC values from 0.5 μM to 8 μM. Vancomycin had MIC values of 0.5 μM to 8 μM and MBC values of 0.5 μM to 4 μM. The findings were classified into Gram-positive and Gram-negative strains and are showcased in Table 2. The data was achieved using a bacterial concentration of 1.5 × 10^5^ CFU per ml.

**Fig. 3:**
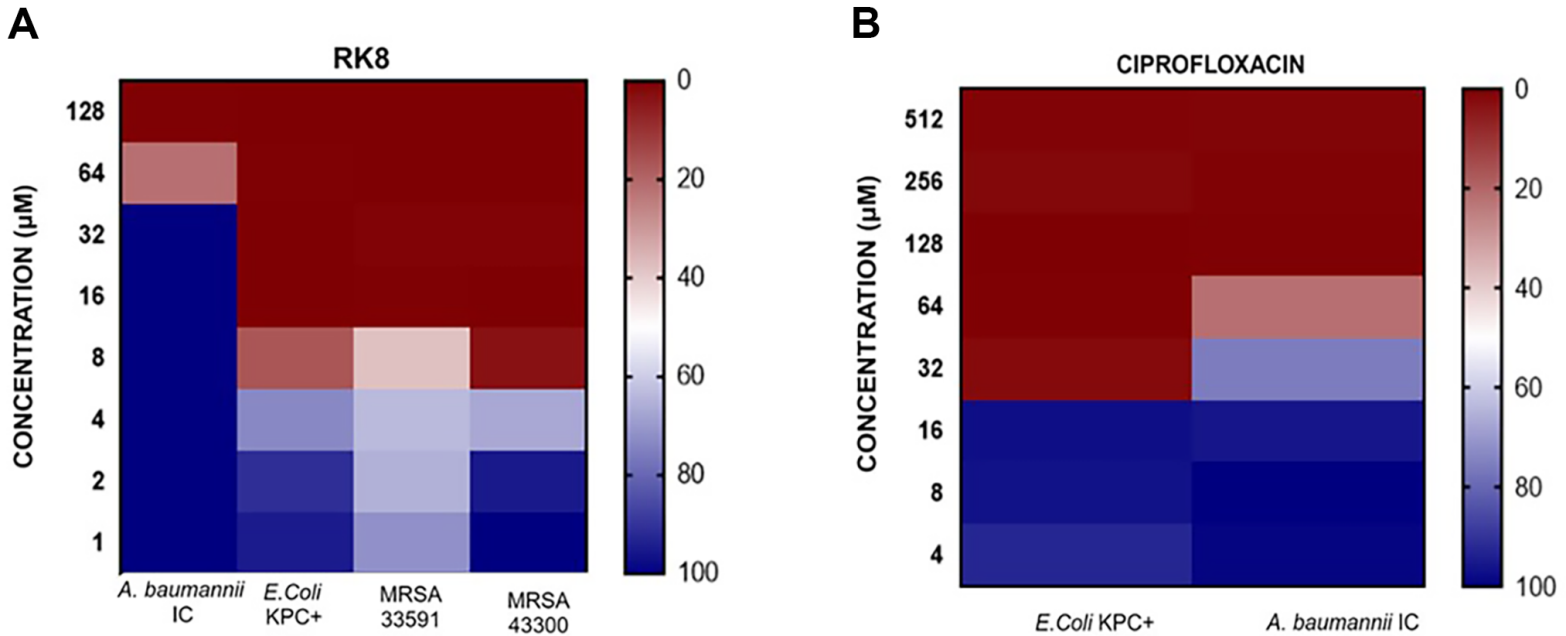
Heatmap graph showing the MICs of RK8 (A) and ciprofloxacin (B) for resistant bacteria, evaluated with an initial density of bacteria corresponding to 1.5 × 10^5^ CFU/ml. Darker red represents growth inhibition and was considered to establish the MIC; lighter shades reveal partial growth inhibition, while dark blue shades show microbial growth.

Following the detection of antibacterial activity against susceptible strains, we evaluated the effectiveness of RK8 against multi-resistant bacterial strains. A heat map was used to improve the clarity of RK8's antibacterial activity data, making it easier to interpret and present the peptide's efficacy against different bacterial strains and concentrations (Fig. 3A and 3B).

The heat map primarily evaluates the effectiveness of RK8. In this context, it is possible to denote that for *A. baumannii* IC, a darker shade of red signifies a more pronounced inhibition by RK8, with higher concentrations (located to the right) being more potent. For *E. coli* KPC, dark red reveals high inhibition, while dark blue shows low inhibition. The effect of RK8 on MRSA 33591 varies with concentration. In contrast, for MRSA 43300, darker red denotes higher effectiveness (see Fig. 3A). Fig. 3B illustrates the efficacy of RK8 compared to ciprofloxacin. *E. coli* KPC+ (IC 001812446) has a minimum inhibitory concentration (MIC) of 32 μM, whereas *A. baumannii* (IC 003321216) shows a significantly higher MIC of 128 μM.

In summary, RK8 is more effective against *A. baumannii* IC and MRSA 43300 than *E. coli* KPC and MRSA 33591, which show lower sensitivity. The overall efficacy of RK8 depends on the concentrations tested and the specific characteristics of each bacterial strain.

### RK8 and Ciprofloxacin Synergistic Effect

**Fig. 4:**
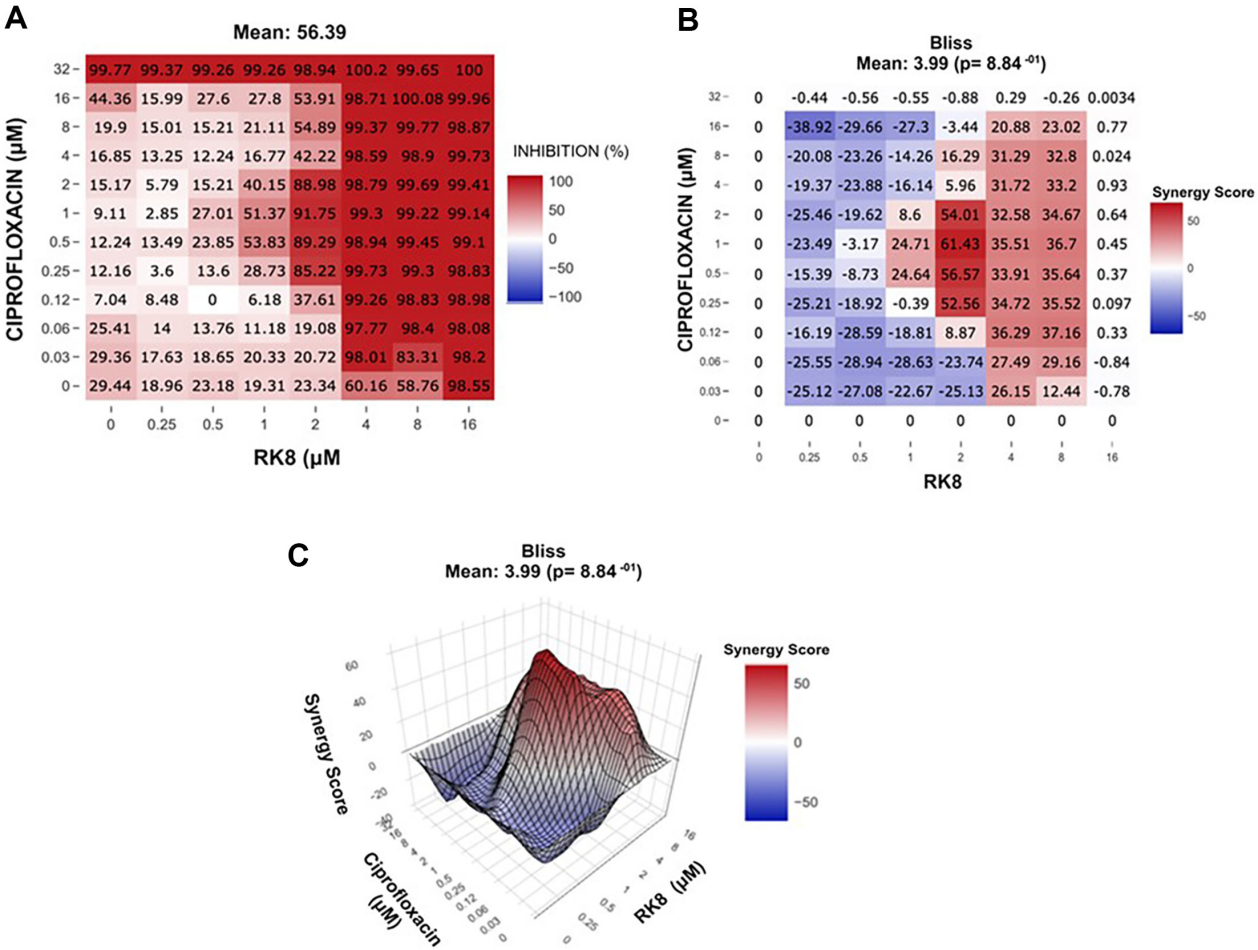
Dose-response inhibition map and Synergy Score for the combination of RK8 and ciprofloxacin against the *E. coli* strain KPC+ IC 001812446.

**Fig. 5:**
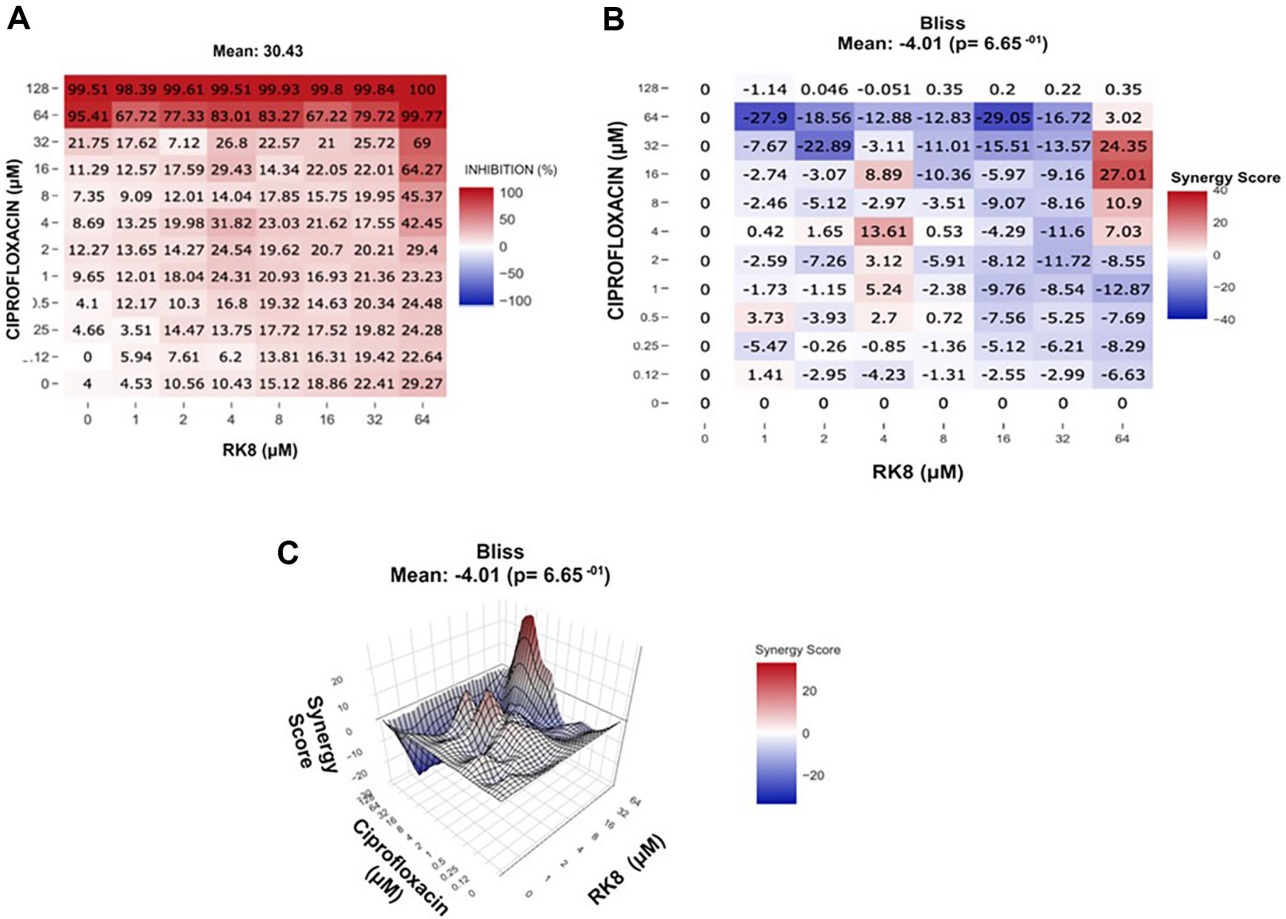
Inhibition dose-response map and Synergy Score for the combination of RK8 and ciprofloxacin against the *A. baumannii* strain IC 003321216.

The RK8 and ciprofloxacin combination showed a Bliss Synergy Score 3.99 against *E. coli* KPC+ IC 001812446. This maintained antimicrobial activity by reducing the concentration of ciprofloxacin by 32-fold and increasing the concentration of the RK8 peptide by 8-fold compared with the MIC (Fig. 4). For *A. baumannii* IC 003321216, the RK8 combination with ciprofloxacin had a -4.01 Bliss Synergy Score value (Fig. 5). This result excludes the possibility of a synergistic effect between the two compounds for the *A. baumannii* strain IC 003321216.

### Antibiofilm Activity

**Fig. 6:**
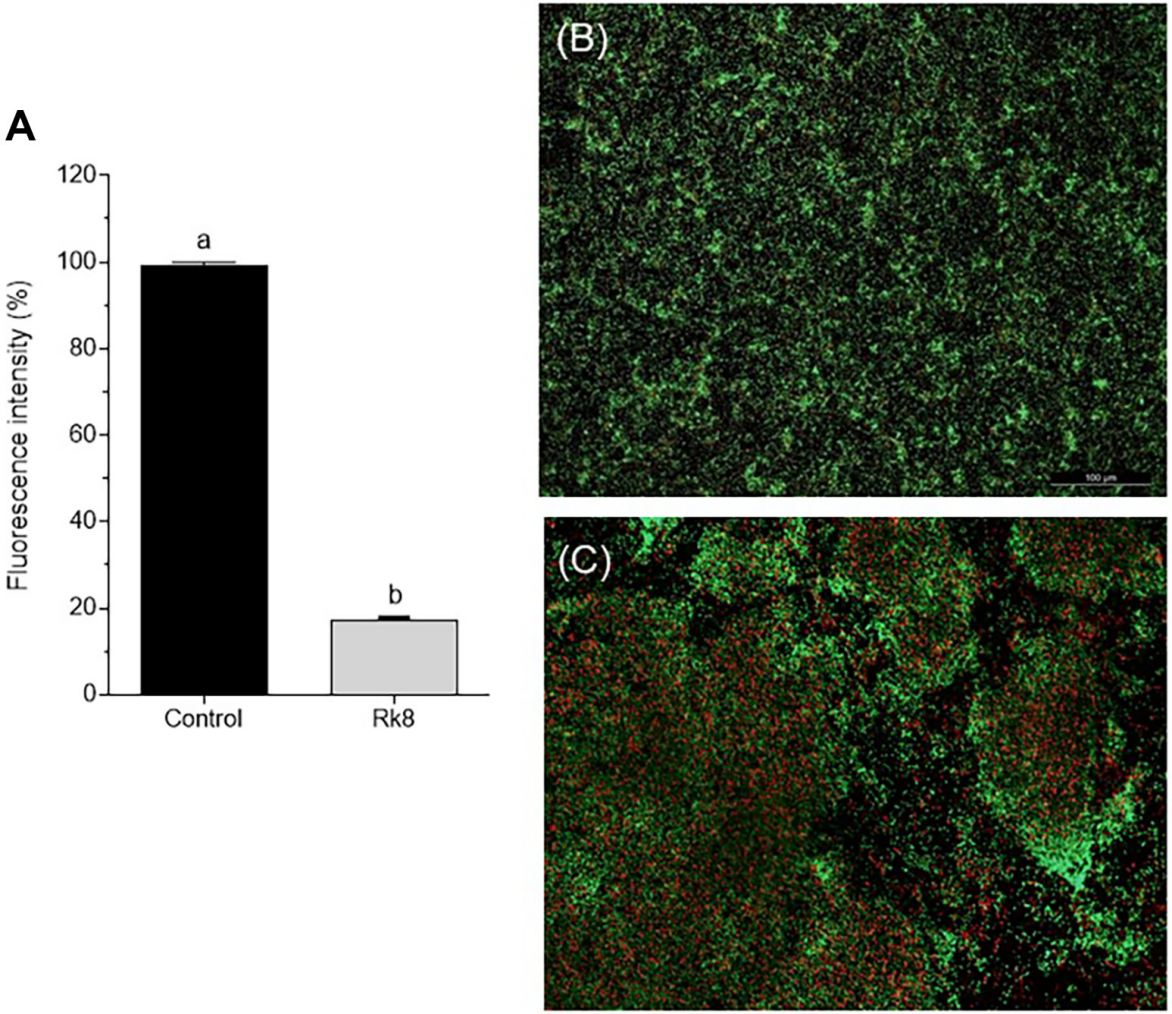
Effect of RK8 on mature biofilm of *A. baumannii* IC 003321216. Viable cells are stained in green, and non-viable cells are stained in red. Image of the control slide without treatment with RK8 in (**A**) and slide with biofilm treated with RK8 in (**B**). *P* < 0.0001 compared with cells treated with control.

**Fig. 7:**
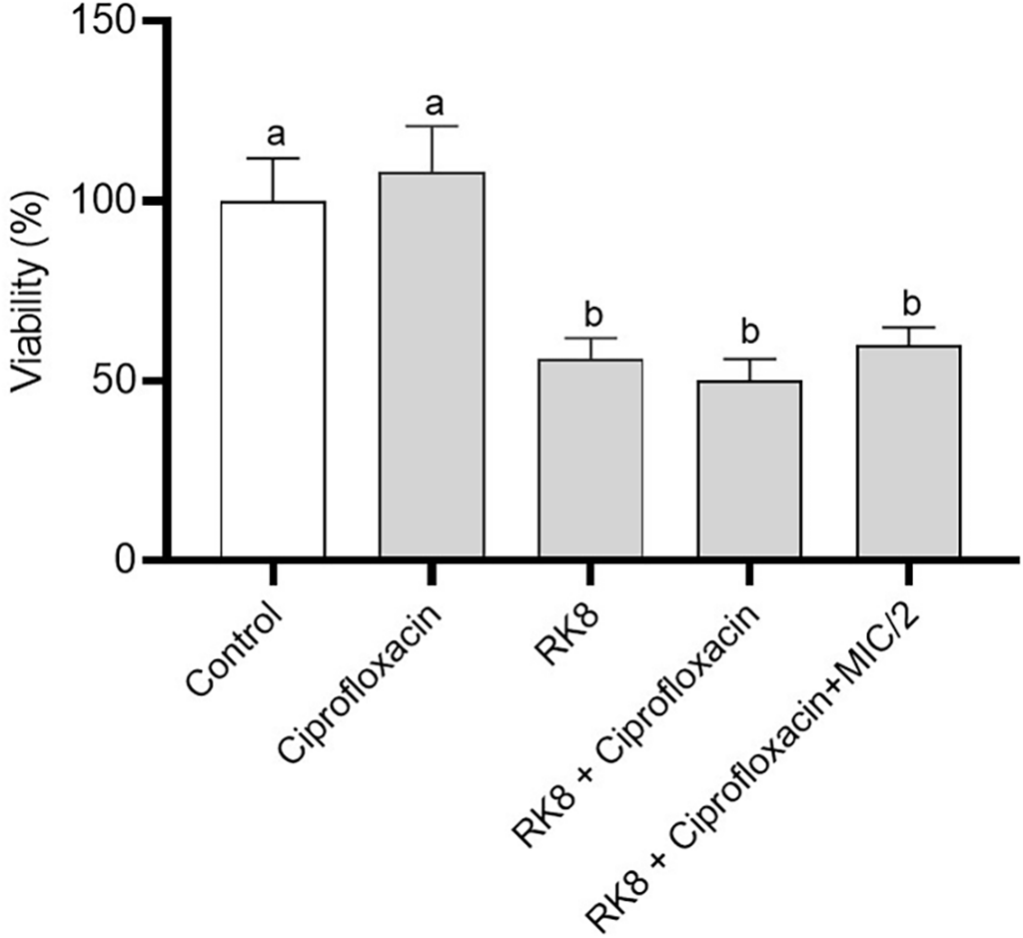
Viability of bacterial cells in mature biofilm of *A. baumannii* IC 003321216, in the presence of RK8 (A) and ciprofloxacin (B). *P* < 0.0001 compared with cells treated with control.

The bacterial eradication biofilm assay was performed with the *A. baumannii* strain IC 003321216. RK8 reduced the *A. baumannii* biofilm mature mass by 38% at MIC, affecting the biofilm structure (Figs. 6 and 7). Ciprofloxacin increased *A. baumannii* biofilm formation by 12% when administered alone at MIC value. No significant difference was observed in the antibiofilm activity of RK8 when administered alone compared to its combination with ciprofloxacin at MIC and MIC/2.

The fluorescence microscopy images (Fig. 6) represent the biofilm viability assay. After exposure to RK8 at the minimum inhibitory concentration (MIC), the biofilm displayed red areas revealing cell death and green areas signifying cell viability. Image analysis using Image J revealed an 82.5% reduction in cell viability. The image shows structural damage to the biofilm, with an irregular distribution of bacteria suggesting significant damage.

### Membrane Permeability

**Fig. 8:**
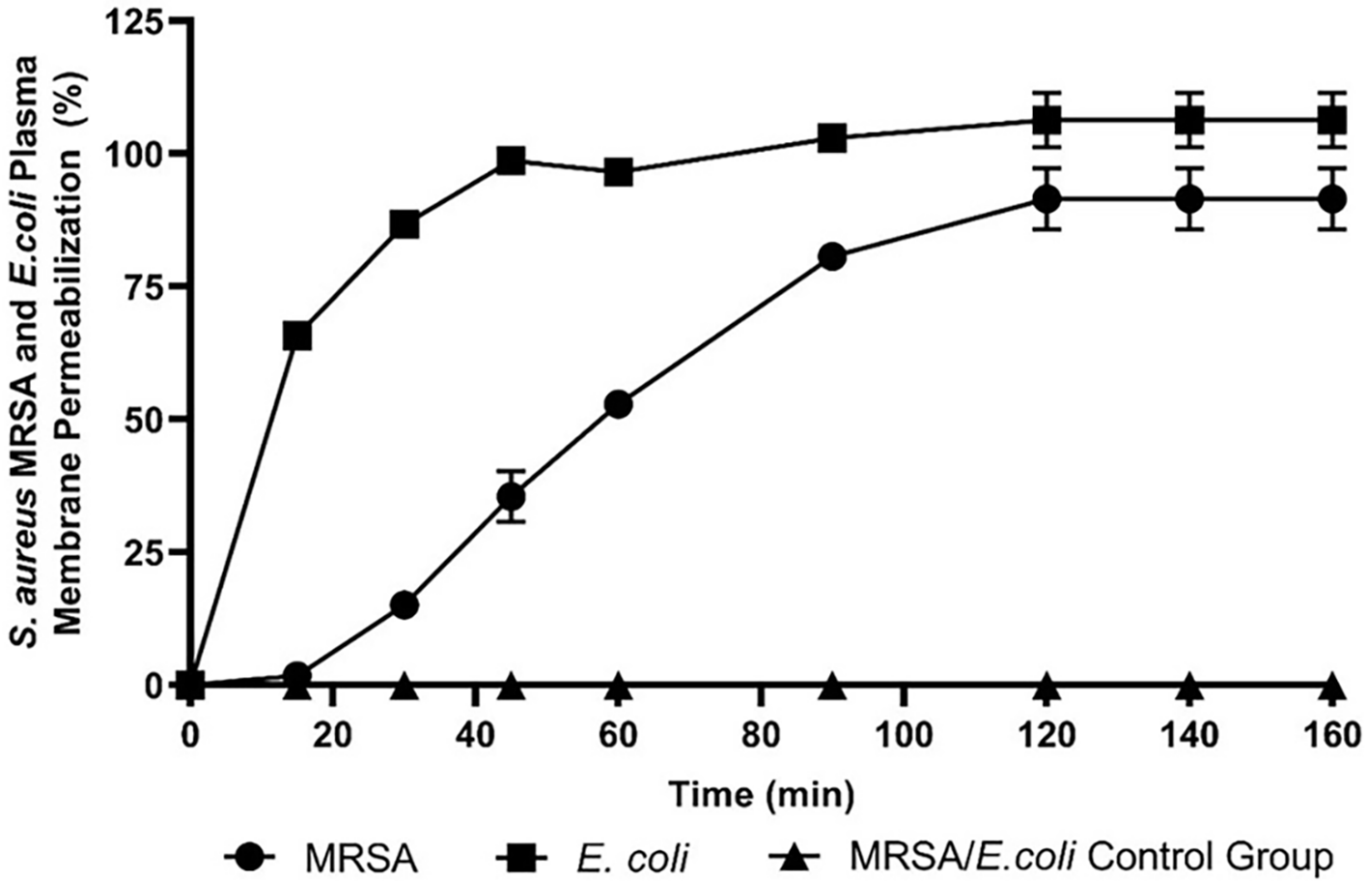
Absorption of Sytox Green by *E. coli* (ATCC 35218) and MRSA (ATCC 43300) treated with RK8 in MIC. Sytox green uptake was measured during 120 min of incubation with RK8.

To verify whether the RK8 mechanism against bacterial cells involves damage to the cell membrane, we performed the assay with the fluorophore Sytox green, a bacterial membrane damage indicator. Fig. 8 shows that the peptide has an action mechanism that involves membrane damage. However, it acts differently on Gram-negative and Gram-positive membranes. For *E. coli*, fluorescence rapidly increased, with 65% membrane permeation from 15 min onwards, reaching a plateau close to 100% from 1 h onwards. A damage mechanism followed a linear pattern for MRSA, requiring two hours to achieve 100% damage. Also, the time it took for Sytox Green to permeate was equal to the time needed to decrease the area under the stability test curve. This observation shows that the peptide exerts its action on the bacterial membrane before undergoing degradation by peptidases.

### Hemolytic Activity

**Fig. 9:**
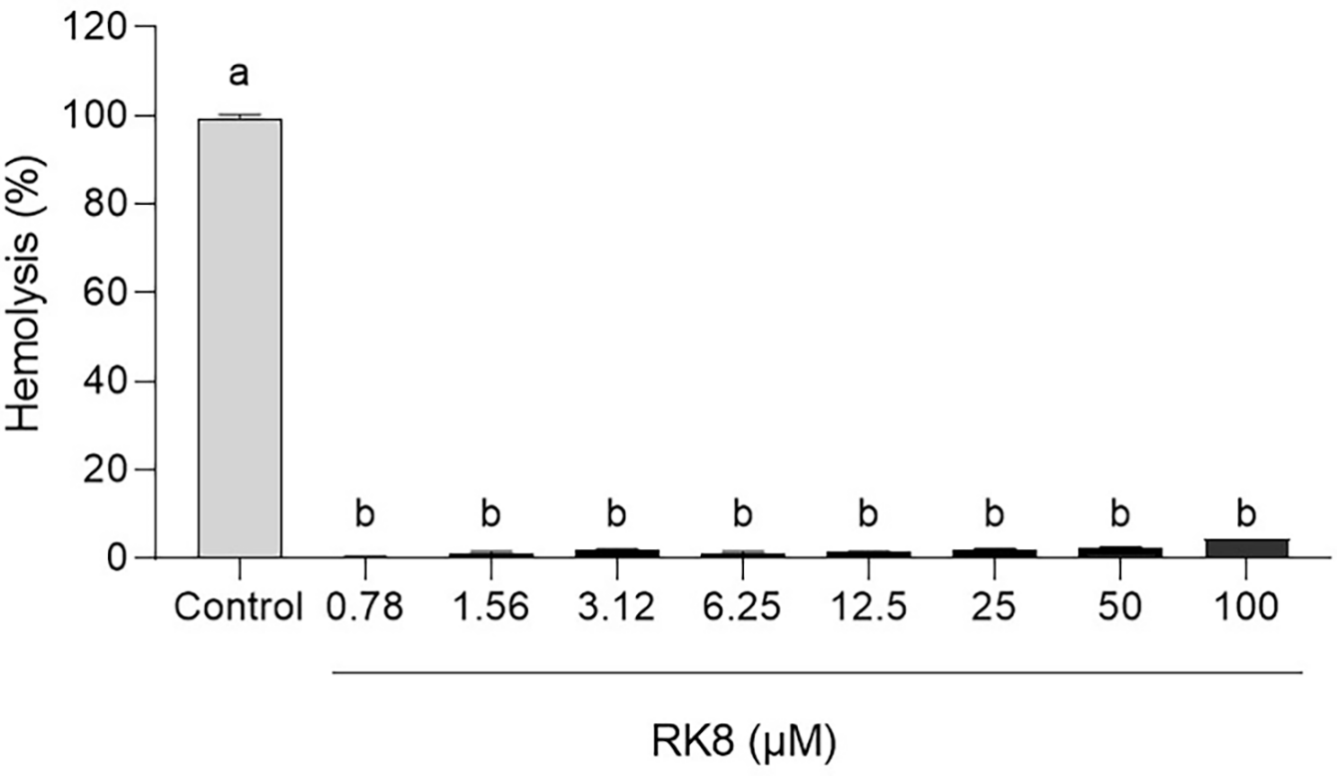
Percentage of hemolysis in the presence of the RK8 peptide at concentrations collected from serial dilution starting at 100 μM. *P* < 0.0001 compared with cells treated with control.

The RK8 hemolytic activity was evaluated against human erythrocytes, where the peptide maximum concentration was 100 μM, performing serial dilution up to 0.78 μM. The RK8 peptide induced approximately 4.6% hemolysis at the initial concentration, having even lower percentages in the MIC bacterial strains tested values (Fig. 9).

### Cell Viability

**Fig. 10:**
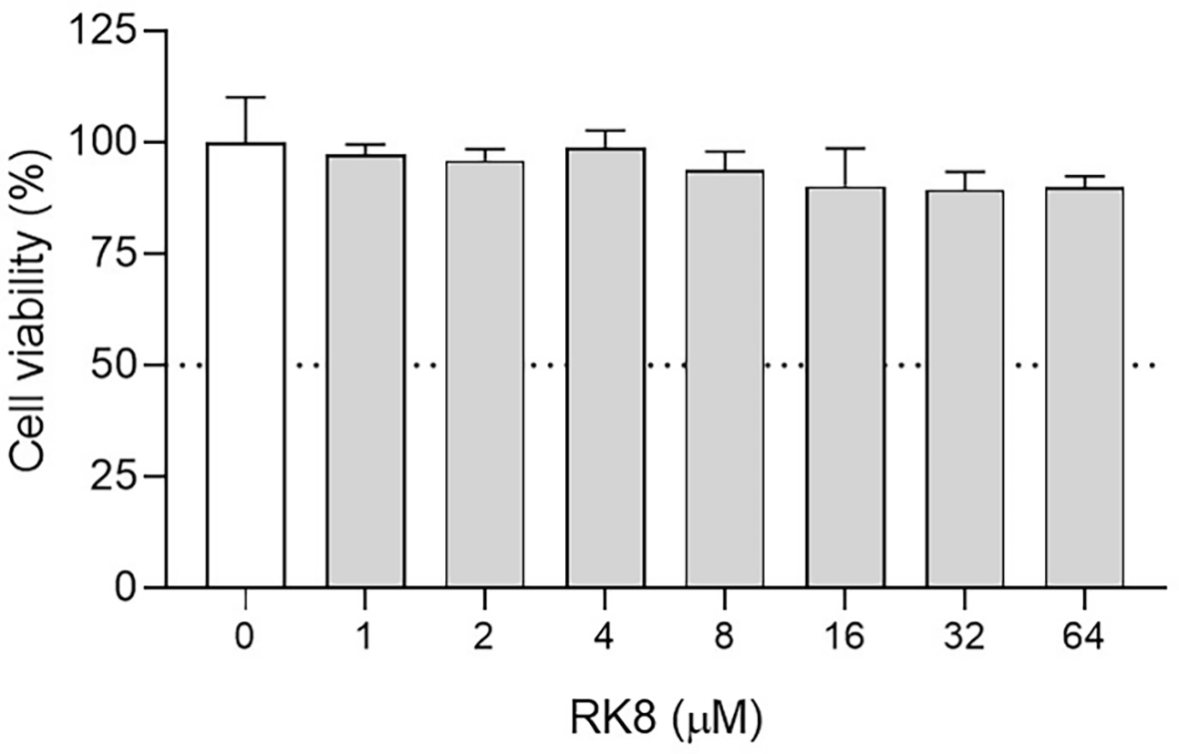
Cell viability test of RAW 264.7 murine macrophages in the presence of the RK8 peptide. Values are means ± SD of three repetitions. *P* > 0.0001 compared with cells treated with the control vehicle.

The cytotoxic effects of RK8 against mammalian cells were evaluated using RAW 264.7 murine macrophages in serial dilution starting at a concentration of 64 μM. At the initial peptide concentration, macrophages maintained their cell viability at 90.1% after 24 h of incubation (Fig. 10). At the concentrations tested, the toxicity index (IC_50_), which is the concentration capable of inducing the death of 50% of cells, was not reached. These results denote that RK8 exhibits low cytotoxicity.

### Acute in vivo Toxicity in *G. mellonella* Assessment

**Fig. 11:**
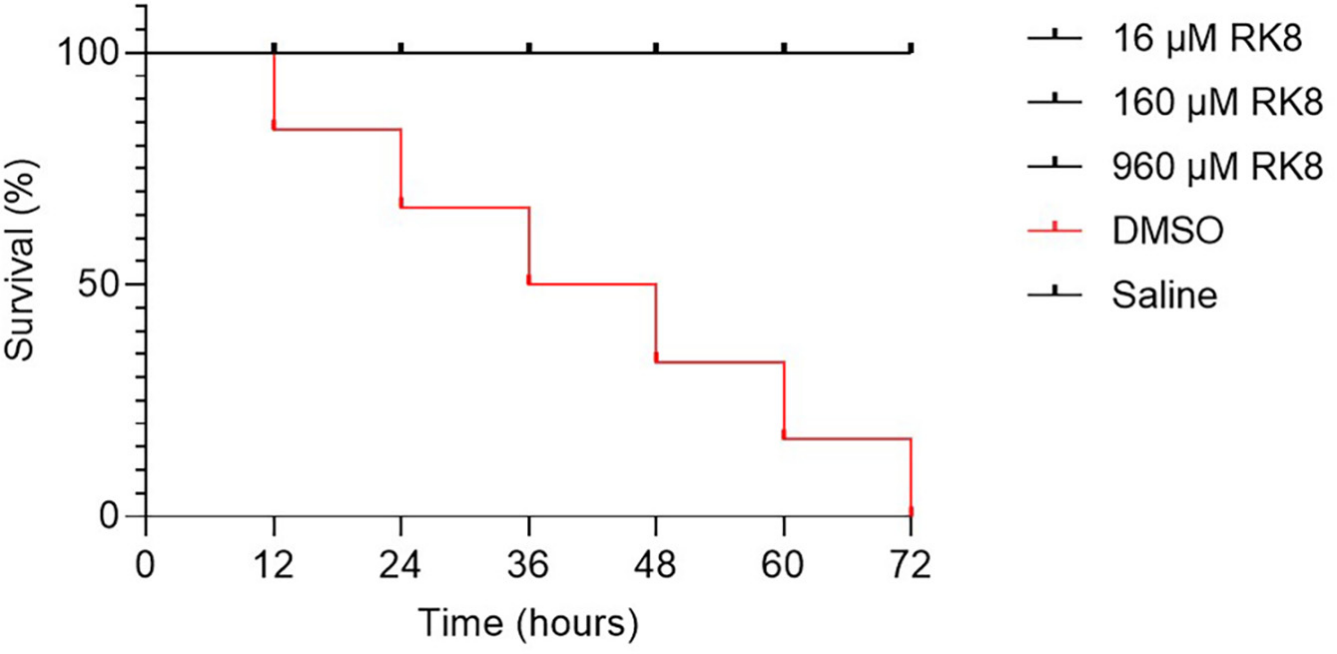
The number of dead *G. mellonella* larvae from 0 h to 72 h was treated with samples at concentrations of 16 μM, 160 μM, and 960 μM (*n* = 10).

As shown in Fig. 11, during the 72 h of the experiment, none of the RK8 concentrations promoted the death of *G. mellonella* larvae, suggesting that the peptide has safe administration.

### Peptide Stability Test

**Fig. 12:**
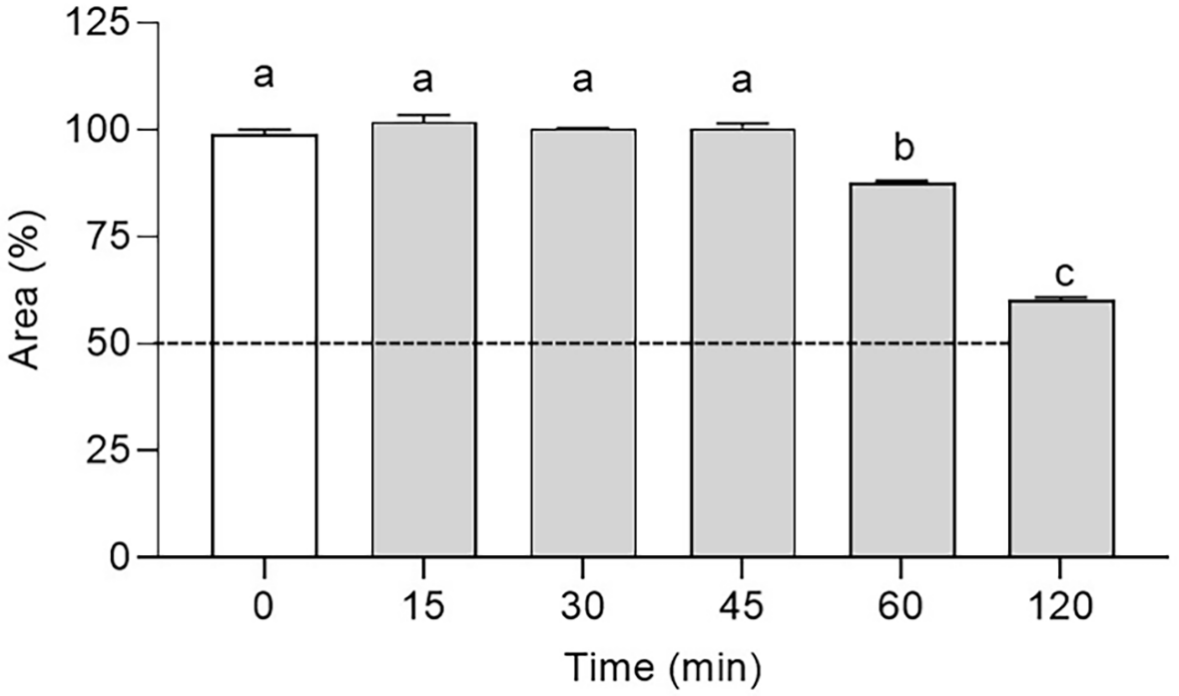
Resistance to degradation of the RK8 peptide incubated in a solution containing 25% FBS. The data represent an assay performed in triplicate, and the percentage of area over the curve was calculated using the Chromeleon Chromatography Data System (CDS) 7 software (Thermo Fisher Scientific). *P* > 0.0001 compared with cells treated with the control vehicle.

After incubating with fetal bovine serum (FBS) at various time intervals, we observed the stability effect of RK8 (Fig. 12). At time zero, 100% of the area under the RK8 peak curve was determined, and this area was used as a parameter to assess the other time intervals. During the first 45-min incubation with 25% FBS, the total area analysed, corresponding to the intact structure of RK8, was unaffected. After 60 min of incubation, we calculated a 12.2% reduction in the area under the curve. Over 120 min, there was a 40% decrease in the overall area, demonstrating RK8's ability to sustain a high level of stability against FBS.

## Discussion

The peptide RK8 comprises 8 amino acid residues, classifying it as an ultrashort peptide belonging to the subfamily of antimicrobial peptides. This specific group offers multiple economic benefits because of its ease of synthesis and purification, resulting in resource and time savings for the synthesis process [20]. The mode of action of the ultrashort cationic peptides is not entirely understood. Recent studies mention that they penetrate phospholipid bilayers, disrupting essential processes such as respiration and cell wall biosynthesis [21]. As mentioned, shorter peptides adopt an extended conformation, which may be rich in one or more specific amino acids. Their mechanism of action is associated with efficiency in internalisation and membrane composition [22].

Boman index calculates the potential interaction of the peptide with membranes (protein-membrane) based on the amino acid sequence. RK8 peptide exhibits a Boman index of 4.93 kcal/mol, with values greater than 2.48 kcal/mol, demonstrating a high potential for binding to biological membranes [23]. The RK8 sequence repeats three amino acids: arginine and lysine, which possess a positive charge, and tryptophan, a nonpolar aromatic amino acid. This combination creates regions with both hydrophobic and hydrophilic properties, facilitating interactions with bacterial membranes. Including mostly positive residues increases the peptide's net electrical charge, enhancing the likelihood of electrostatic attraction between the AMP and the predominantly anionic microbial membrane [24]. Research suggests that in AMPs, there is an approximate 50% increase in the presence of essential amino acids like Arginine and Lysine compared to the overall genomic content. Conversely, acidic amino acids exhibit a notable reduction, approximately 75% less than expected. AMPs commonly contain hydrophobic amino acids, demonstrating their prevalence in these peptides. These findings are consistent because they are designed using simplified sets of amino acids, predominantly comprising essential and hydrophobic residues. Specifically, straightforward cationic/hydrophobic peptides, such as those containing Arginine and Tryptophan, demonstrate notable antimicrobial efficacy [25].

CD analysis reveals that the RK8 peptide lacks a structured conformation, meaning its amino acid residues do not form regular secondary structures. These findings categorise it as an extended AMP, a characteristic often observed in peptides rich in amino acids such as Arginine (Arg), Tryptophan (Trp), or Proline (Pro). Notably, Trp and Arg residues are prevalent in peptides with fewer than 15 residues, exemplified by the active antimicrobial fragments RRWQWR and RAWVAWR from bovine lactoferricin and human lysozyme, respectively. Screening of combinatorial peptide libraries uncovered potent broad-spectrum activity in the hexameric sequence RRWWRF [26].

In Gram-negative bacterial strains, RK8 demonstrated consistent MIC and MBC values, exhibiting a bactericidal effect within concentrations ranging from 0.5 to 4 μM against *E. coli*, *P. aeruginosa*, and *A. baumanii*. Conversely, for the Gram-positive *S. aureus* strain, RK8 displayed an MIC of 0.5 μM, exerting bacteriostatic effects at this concentration while achieving a bactericidal effect at one μM, as shown by the MBC value. Comparatively, other tryptophan-rich peptides like Indolicidin and Tritropcytin exhibit an MIC of 10 μM against *E. coli*, with Tritrepticin showing a similar MIC value for *S. aureus* [27]. The RK8 peptide exhibited an MIC of 0.5 μM against the *P. aeruginosa* strain. Contrastingly, the synthetic peptide Adepamycin comprises bacterial activity against *P. aeruginosa* at a concentration of 2.8 μM [28]. It can be deduced that the design of RK8 has contributed to its antimicrobial efficacy, positioning it favourably alongside various antimicrobial peptides (AMPs) recognised for their potent antimicrobial activity.

In clinical isolates exhibiting resistance, RK8 displayed its most favourable MIC value against *E. coli* KPC+ (001812446), registering at 16 μM. Multidrug-resistant MRSA ATCC 43300 exhibited the same MIC value. Addressing infections caused by carbapenem-resistant strains presents a significant clinical hurdle, given their resistance to β-lactam antibiotics, typically reserved as a last line of defence [29]. Methicillin, a synthetic beta-lactam antibiotic, has seen the emergence of *S. aureus* strains resistant to its effects over time, evaluated the antimicrobial activity of 30 AMPs against MRSA, with MIC values ranging from 3.1 to 92 μM [30]. Ciprofloxacin, the third most prescribed antibiotic, was also assessed for its MIC against resistant clinical isolates, yielding values of 32 and 128 μM for *E. coli* KPC+ (001812446) and *A. baumannii* (003321216), respectively. Because of its widespread use, bacteria have resisted ciprofloxacin [31, 32].

The treatment of resistant pathogens often involves multiple antibiotic combinations to achieve a synergistic effect. However, this approach remains contentious because of the heightened toxicity risk, organ damage, and the potential for selecting resistant strains. In vitro synergy evaluations typically utilise checkerboard titration, although some studies also employ animal models. According to Pletzer *et al*. [33], combining AMPs with antibiotics in vivo has shown the potential to enhance the treatment outcomes of multidrug-resistant infections. For instance, in their study, a combination of ciprofloxacin with the synthetic peptide DJK-5 resulted in a 245-fold reduction in *P. aeruginosa* bacterial load and a 10.3-fold decrease in *E. coli*, representing a 2.9-fold improvement compared to individual monotherapies. In vitro, RK8 at 2 μM in combination with ciprofloxacin at 1 μM inhibited 91.75% of the growth of the clinical isolate *E. coli* KPC+ (001812446). This outcome signifies an 8-fold decrease in RK8 concentration and a 32-fold decrease in ciprofloxacin concentration compared to their respective individual MIC values for *E. coli* KPC+ (001812446).

RK8 disrupted approximately 40% of mature *A. baumannii* biofilm at the MIC level. When combined with ciprofloxacin, RK8 showed a comparable outcome, suggesting a synergistic effect on antibiofilm activity. Notably, ciprofloxacin alone exhibited no impact on the targeted biofilm formation. *A. baumannii*, known for colonising medical equipment surfaces like urinary catheters to form a biofilm, contributes to recurrent and persistent patient infections [34]. WLBU-2, a synthetic cationic peptide, and LL-37, a naturally occurring antimicrobial peptide, underwent testing for efficacy against a range of ESKAPE pathogens. At concentrations equivalent to one-third of the MIC, they manifest 90% inhibition of biofilm formation compared to antibiotics like tobramycin, ciprofloxacin, ceftazidime, and vancomycin in MIC [35]. A hydrogel formulation containing K-11, a melittin hybrid peptide, cecropin A1, and magainin-2 showed promising wound healing capabilities in a murine infection model infected with *A. baumannii*, suggesting its potential as a topical anti-inflammatory therapeutic agent for infectious diseases [36].

The outer membrane of Gram-negative bacteria involves an asymmetric bilayer comprising phospholipids and lipopolysaccharides (LPS). The initial interaction between AMPs and the membrane of Gram-negative bacteria involves electrostatic attractions between the positively charged peptide and the negatively charged LPS of the outer membrane, ultimately resulting in membrane disruption [37, 38]. To investigate the potential mechanism of action of RK8, we assessed whether it induces damage to bacterial membranes using the Sytox green reagent. Sytox green is a DNA intercalator that cannot penetrate intact membranes. By conducting fluorescence microscopy studies, we aimed to detect the leakage of intracellular contents into the extracellular environment [39]. In this study, we aimed to observe an increase in fluorescence when bacteria were exposed to the peptide, illustrating potential damage to microbial membranes. Our results revealed that membrane permeation occurred more rapidly in Gram-negative strains than in Gram-positive ones. The time required for Sytox green permeation was shorter than the interval needed to observe a reduced area under the stability test curve. This suggests that the peptide's action on bacterial membranes theoretically precedes its degradation by peptidases.

When designing a peptide, it is crucial to consider the potential cytotoxic effects on eukaryotic cells. The relationship between structure and activity plays an essential role in determining the selectivity of the peptide towards its target cells [40]. Eukaryotic plasma membranes primarily contain neutrally charged or zwitterionic lipids, unlike bacterial membranes containing anionic components. Therefore, the interaction of AMPs with other cells is generally considered selective [41]. The concentrations tested proved entirely safe, with negligible hemolysis observed even at concentrations much higher than the MIC and MBC. The presence of RK8 did not significantly affect the cell viability of murine macrophages if its cytotoxicity index (IC_50_), signalling the concentration capable of inducing cell death in 50% of the cells, could not be determined in the assay, thus suggesting potential pharmacological safety.

There is a growing trend towards minimising the use of mammals in vivo testing, leading to increased reliance on in vitro toxicity data derived from cell cultures. While in vitro models are useful for studying certain aspects of compound metabolism, such as absorption rates, biotransformation, distribution, and excretion, they may not fully replicate the complexities of these processes in the human body. *G. mellonella* larvae have emerged as an ethical alternative for research, as they reflect certain complexities present in mammals [42]. For instance, *G. mellonella* larvae possess an innate immune system that is functionally and structurally similar to mammals [43, 44]. In the present study, all treated larvae exhibited 100% survival across all tested concentrations during the experimental period, suggesting the non-toxic nature of RK8 in an experimental model, even at concentrations up to 60 times higher than those required to eradicate multidrug-resistant *E. coli* KPC+ (001812446).

The peptide's stability in blood is critical for its in vivo antimicrobial effectiveness, demanding prolonged circulation at high blood concentrations. Peptides' in vivo stability in blood can be simulated by assessing their stability in serum or plasma in vitro [19]. In this study, the RK8 peptide was tested in the presence of fetal bovine serum to determine its half-life against enzymatic degradation. Nguyen *et al*. [45] investigated the stability of several short AMPs rich in Trp and Arg in the presence of 25% human serum. Peptides with free C- and N-terminal ends, such as Lfc1 and Com1, underwent rapid degradation, with a half-life of 30 minutes and complete degradation observed after 2 h of testing. Another study focused on the antimicrobial peptide Jelleine-I and four analogues in the presence of 25% mouse serum. During halogenation, Jelleine-I and two analogues (FJI and Cl-JI) maintained 10% structural integrity after a 2-h incubation period to enhance AMPs' antimicrobial effectiveness and durability. In contrast, the analogues Br-JI and IJI exhibited more excellent resistance, with 50% of the peptide structure remaining intact after 2 h [46]. Compared to these studies, RK8 displayed less degradation over the same testing period and remained stable for a longer duration. Further investigation will determine whether the proximity of Trp residues to Arg and Lys residues can hinder enzyme access to these residues, increasing their half-life.

## Conclusion

The findings from this study underscore the efficacy of the design approach employed in creating RK8, yielding a novel ultrashort and extended AMP. RK8 exhibited significant efficacy against Gram-positive and Gram-negative bacteria, including multidrug-resistant clinical isolates. These results suggest that the bacterial plasma membrane may serve as a target for RK8, acting before its degradation by peptidases found in blood plasma. In vitro toxicity assessments against erythrocytes and macrophages and in vivo experiments with *G. mellonella* suggest the safety of RK8 administration. Studies revealed a synergistic effect of RK8 in combination with ciprofloxacin, reducing the concentrations required to achieve antimicrobial activity against *E. coli* KPC+. The RK8 demonstrated antibiofilm efficacy, disrupting 38% of mature *A. baumannii* biofilm and compromising biofilm structure and bacterial viability. Thus, RK8 is a promising avenue for developing a new antimicrobial agent.

## Figures and Tables

**Table 1:** Physicochemical characteristics of the RK8 peptide attained from the APD3 server.

Sequence	Total net charge	Boman Index (Kcal/mol)	pI	Molecular weight (Da)	Hydrophobic reason (%)
RKWRKWWK	+5	4.93	12.51	1.273,54	38%

**Table 2:** MIC and MBC values, in μM, of Gram-positive and Gram-negative strains were evaluated with an initial density of bacteria corresponding to 1.5 × 10^5^ CFU/ml.

Microorganisms (1.5 × 10^5^ CFU/ ml)							
	Bacteria	RK8		Ciprofloxacin		Vancomycin	
		MIC (μM)	MBC (μM)	MIC (μM)	MBC (μM)	MIC (μM)	MBC (μM
Gram-positive	*Staphylococcus aureus* (MRSA) ATCC 33591	8	8	4	8	1	1
	*Staphylococcus aureus* (MRSA) ATCC 43300	16	8	2	2	1	0.5
	*Staphylococcus saprophyticus* ATCC 29970	>64	64	1	1	0.5	1
Gram-negative	*Escherichia coli* KPC+ 001812446	16	8	32	1	0.5	0.5
	*Escherichia coli* ATCC 35218	>64	2	1	2	8	1
	*Acinetobacter baumannii* IC 003321216	64	4	128	1	4	1
	*Acinetobacter baumannii* ATCC 19906	>64	4	1	1	4	1
	*Pseudomonas aeruginosa* ATCC 27853	>64	64	0.5	8	0.5	1
	*Klebsiella pneumoniae* ATCC 700603	>64	64	12	1	8	1

MIC, Minimum Inhibitory Concentration; MBC, minimum Bactericidal Concentration
